# Prevention of congenital chagas disease by trypanocide treatment in women of reproductive age: A meta-analysis of observational studies

**DOI:** 10.1371/journal.pntd.0012407

**Published:** 2024-09-05

**Authors:** Francisco Cezar Aquino de Moraes, Maria Eduarda Cavalcanti Souza, Lucca Dal Moro, Isabelle Batista Donadon, Emanuele Rocha da Silva, Dilma do Socorro Moraes de Souza, Rommel Mario Rodríguez Burbano

**Affiliations:** 1 Federal University of Pará, Belém, Brazil; 2 University of Pernambuco, Recife, Brazil; 3 Universidade Maimonides, Buenos Aires, Argentina; 4 Gaspar Vianna State public Hospital, Belém, Brazil; 5 Hospital Ophir Loyola, Belém, Brazil; University of Texas at El Paso, UNITED STATES OF AMERICA

## Abstract

**Background:**

Maternal-foetal transmission of Chagas disease (CD) affects newborns worldwide. Although Benznidazole and Nifurtimox therapies are the standard treatments, their use during pregnancy is contra-indicated. The effectiveness of trypanocidal medications in preventing congenital Chagas Disease (cCD) in the offsprings of women diagnosed with CD was highly suggested by other studies.

**Methods:**

We performed a systematic review and meta-analysis of studies evaluating the effectiveness of treatment for CD in women of childbearing age and reporting frequencies of cCD in their children. PubMed, Scopus, Web of Science, Cochrane Library, and LILACS databases were systematically searched. Statistical analysis was performed using Rstudio 4.2 using DerSimonian and Laird random-effects models. Heterogeneity was examined with the Cochran Q test and I^2^ statistics. A p-value of <0.05 was considered statistically significant.

**Results:**

Six studies were included, comprising 744 children, of whom 286 (38.4%) were born from women previously treated with Benznidazole or Nifurtimox, trypanocidal agents. The primary outcome of the proportion of children who were seropositive for cCD, confirmed by serology, was signigicantly lower among women who were previously treated with no congenital transmission registered (OR 0.05; 95% Cl 0.01–0.27; p = 0.000432; I^2^ = 0%). In women previously treated with trypanocidal drugs, the pooled prevalence of cCD was 0.0% (95% Cl 0–0.91%; I^2^ = 0%), our meta-analysis confirms the excellent effectiveness of this treatment. The prevalence of adverse events in women previously treated with antitrypanocidal therapies was 14.01% (95% CI 1.87–26.14%; I^2^ = 80%), Benznidazole had a higher incidence of side effects than Nifurtimox (76% vs 24%).

**Conclusion:**

The use of trypanocidal therapy in women at reproductive age with CD is an effective strategy for the prevention of cCD, with a complete elimination of congenital transmission of *Trypanosoma cruzi* in treated vs untreated infected women.

## Author summary

Chagas Disease (CD), a neglected tropical disease (NTD), is a global public health issue with an estimated 70 million people being at risk of infection. Congenital transmission is currently the main mode of transmission of *T*. *cruzi* in both endemic and non-endemic countries. Most women of gestational age in endemic countries are chronically infected, having acquired the infection during infancy. Vertical transmission happens in 5% of them, and thus approximately 9,000 newborns infected with CD in Latin America are born annually. The available data on preventive treatments for pregnant women and the rate of congenital transmission is still limited with existing epidemiological information predominantly based on estimations. Therefore, our meta-analysis confirms the evidence for the use of trypanocidal agents in infected women of reproductive age to prevent congenital *Trypanosoma cruzi* (Tc) infection.

## Introduction

Chagas disease is an infection caused by Trypanosoma cruzi and is endemic in 21 countries in the Americas. It is estimated that 6 million people worldwide are infected with the protozoan [1–3]. During pregnancy, high maternal parasitemia is thoroughly associated with congenital transmission [4,5].

Congenital transmission of Chagas disease occurs through the hematogenous transplacental route and can occur in each pregnancy of infected women [6]. Although most cases are asymptomatic or mild, the clinical symptoms of the congenital infection are highly varied, including miscarriages, premature birth, low birth weight, stillbirths, and clinical manifestations of the disease at birth, such as respiratory distress, hepatosplenomegaly, anemia, myocarditis, meningoencephalitis, and megaviscera [4, 7–10]. Congenital transmission in endemic countries, when compared with countries free of vector transmission, is twice as likely to be congenital, reaching 4.7% and 2.7%, respectively [3].

Currently, T. cruzi infection is globally distributed, and its presence in non-endemic countries has been associated with migration, especially from Latin America [11,12]. In the Americas, approximately 9,000 newborns are infected during pregnancy. It is estimated that the prevalence of pregnant women infected with T. cruzi ranges from 1% to 40%, and approximately 1,124,930 Latin American women aged between 15 and 44 years are infected with T. cruzi [1,7,13].

The World Health Organization (WHO) does not recommend the use of Benznidazole and nifurtimox in pregnant women, as the teratogenic risks of the available antiparasitic therapy are not well-known, and the risk of adverse reactions is high in adults [5]. Therefore, trypanocidal treatment of reproductive-aged women infected may be effective in reducing vertical transmission in future pregnancies [5].

Therefore, this systematic review and meta-analysis aims to assess the the effectiveness of treatment with trypanocidal therapy in non-pregnant women in reproductive age for preventing congenital transmission of *Trypanosoma cruzi*.

## Methods

### Protocol and registration

The systematic review and meta-analysis were performed by the Cochrane Collaboration and the Preferred Reporting Items for Systematic Reviews and Meta-Analysis (PRISMA)(S1 and S2 Tables) [14]. The study protocol was registered in the International Prospective Register of Systematic Reviews database-PROSPERO (registration number: CRD42023418819).

### Eligibility criteria

Studies that met the following eligibility criteria were included: (1) observational cohort and case-control studies, (2) non pregnant women ≥18 years of age with use of trypanocidal therapy. We excluded studies with overlapping populations, studies with patients with Diabetes Mellitus, prior toxoplasmosis or HIV infection, immunosuppressed individuals, poor obstetric history, and other comorbidities other than Chagas disease, and studies with no outcomes of interest. Case reports, review articles, opinion articles, technical articles, guidelines, animal studies, and in vitro studies were also excluded.

Thus, we sought to answer whether prior treatment with trypanocidal therapy in non-pregnant women in reproductive age could be effective on preventing congenital transmission of *Trypanosoma cruzi*.

In our meta-analysis, we defined trypanocidal drugs as the agents used to treat Chagas disease. Currently, two drugs with proven effectiveness against T. cruzi are available: benznidazole and nifurtimox [15,16]. The details and characteristics of the drugs included in this study are summarized in S1 Table.

### Search strategy

PubMed, Scopus, Web of Science, Cochrane Library, and LILACS were systematically searched from inception to March 2023. The keywords used in this search were: “Chagas disease”, “trypanosomiasis”, “reproductive age”, “pregnancy”, “trypanocide therapy”, “benznidazole”, nifurtimox”, “congenital Chagas”, and “prevention”. The search strategy with the MeSH terms is more detailed in S3 Table.

This strategy was supplemented by searching the references of the included articles, abstracts, conference reports, and systematic reviews of the literature. Aiming for the inclusion of additional studies, the references of the included articles and systematic reviews of the literature were evaluated and an alert was established for notifications in each database, in case a study corresponding to the consultation carried out was eventually published. Those found in the databases and the references of the articles were incorporated into the reference management software (EndNote, version X7, Thomson Reuters, Philadelphia, USA). We did not use any date or language restrictions in the electronic searches for trials.

Duplicate articles were automatically and manually excluded. Titles and abstracts of identified articles found in the databases were analyzed independently by two reviewers (F.C.A.M. and L.D.M.). Disagreements were resolved by consensus between the two authors and the senior author (F.C.A.M., L.D.M., and R.M.R.B.).

### Data extraction

The following baseline characteristics were extracted: (1) study design; (2) regimen details (choice of medication, dosage, treatment duration); (3) number of women and children; (4) total number of mother-child events; (5) follow-up; and the diagnostic test.

Where available, the full protocol of each study was consulted to verify study objectives, population, and other relevant information regarding study design and conduction. For publications reporting results from the same study, the most recent or complete publication reporting the information of interest was considered.

### Endpoints

The following outcomes of interest were extracted: (1) Congenital transmission of chagas disease in women who have used previous trypanocidal therapy, (2) prevalence of cCD transmission, and (3) adverse events in women.

### Risk of bias assessment

The quality assessment of observational studies was performed using the Newcastle–Ottawa Scale (NOS), in which studies are scored on a 0 to 9 scale according to selection, comparability, and exposure criteria [17]. Two review authors (F.C.M., and L.S.) independently conducted the process of quality assessment. Disagreements were resolved by consensus.

### Statistical analysis

We conducted a proportional meta-analysis pooling the data with the function “and “pool.median”, included in the packages “meta” and “metafor for effectiveness outcomes. The prevalence of Congenital Chagas with 95% confidence interval (CI) from binary analyses was used to assess efficiency factors of trypanocide medication, using the function “metagen”, also included in the “meta” package in R. Cochran’s Q test and I^2^ statistics were used to evaluate heterogeneity; *p*-values inferior to 0.10 and I^2^ > 25% were considered significant for heterogeneity [18]. The Sidik-Jonkman estimator was used to calculate the tau^2^ variance between studies [19]. A DerSimonian and Laird random-effects model was applied to all endpoints [20]. We used RStudio (Posit Software, PBC, version 2022.12.0 + 353) for statistical analysis.

## Results

**Fig 1:**
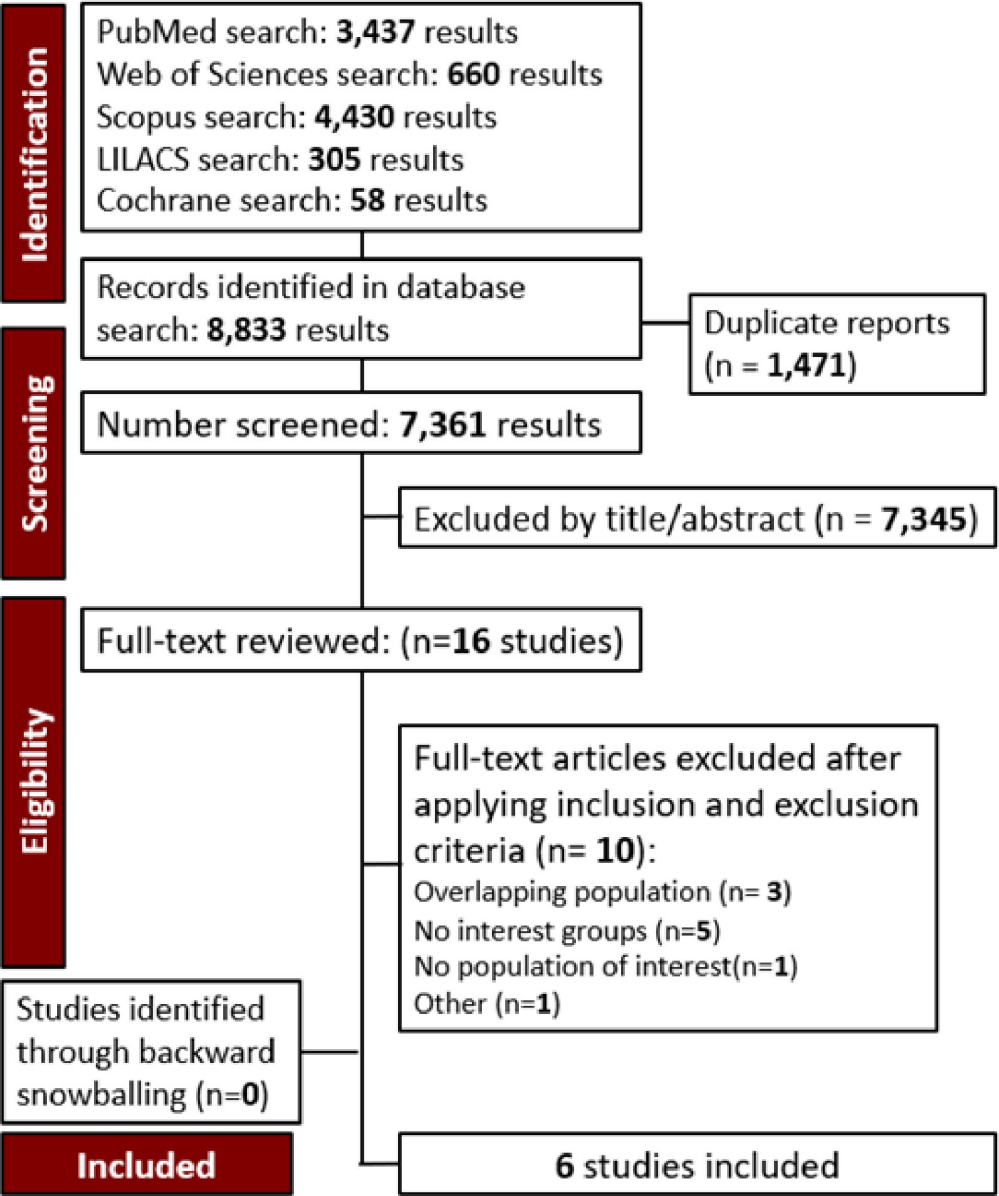
PRISMA flow diagram of study screening and selection.

The selection was detailed in a PRISMA flow diagram (Fig 1). A total of 8,833 references were retrieved in our systematic search. After the removal of duplicate records, and the assessment of the studies based on title and abstract, 8,816 references were excluded and 16 full-text manuscripts were eligible and thoroughly reviewed for inclusion and exclusion criteria. Of these, six observational studies in 16 references satisfied the eligibility criteria and formed the scope of the analysis [21–26]. No Randomized Controlled Trials were as available by the time this study was conducted.

A total of 1,197 patients were analyzed, comprising 742 women infected and 744 children. Study patient baseline characteristics of the included studies are summarized in Table 1. In the overall population, 255 of 742 women of childbearing age received previous therapy. The age of patients ranged from 15 to 45 years old, with a follow-up time that ranged from 3 years to 16,3 to 18,6 years.

**Table 1:** Design and Characterístics of Studies Included in the Meta-analysis.

Author	Mother Age†	Follow-up†	Confirmatory examination of Mother /child	Treatment§	Treatment duration§	N of children	Mother-child pair	Maternal geographical origin
Álvarez [21]	15–45	2–5	Serology	Bzn (5 mg/kg)	30	156	156	NA
Fabbro [22]	15–45	16,3–18,6	Serology (IHA, ELISA)	Bzn (5,16 mg/kg) and Nfx (10 mg/kg)	15–66 (Bzn) and 14–90 (Nfx)	354	354	Argentina; Bolivia; Paraguay; Uruguay
Murcia [23]	15–45	mother (1–9); children (0,6–12 months)	Serology (ELISA, IHA) and PCR	Bzn (5–7 mg/kg) and Nfx (10 mg/kg)	60 (Bzn; Nfx)	160	159	Bolivia; Paraguai; Ecuador; Argentina
Moscatelli [24]	9–34	3	Serology, PCR and ELISA	Bzn (5–7,6 mg/kg) and Nfx (9,4 mg/kg)	19–60 (Bzn; Nfx)	16	15	Argentina
Sosa-Estani [25]	21–29	14	Serology (AIE, IHA, IFI) and PCR	Bzn (6 mg/kg)	60	32	32	Argentina
ALTCHEH [26]	≤18	3	Serology (ELISA, IHA)	Nfx (NA)	30–60	26	26	NA

† Years

§ Day; N: number; Bzn: benznidazole; ELISA: enzyme-linked immunosorbent assay; IHA: Indirect Hemagglutination; Nfx: nifurtimox; NA: not avalaible; PCR: Polymerase chain reaction

The confirmation of Chagas Disease in the female population was determined through laboratory serological tests, these constitute the WHO diagnostic gold standard [5]. The main methods used were: Enzyme-linked immunosorbent assay (ELISA) in 3 studies, Indirect Hemagglutination (IHA) in 3 studies. The diagnostic in children was also performed using ELISA and IHA serological techniques. The diagnosis of congenital Trypanosoma cruzi infection in infants can be done through direct parasitological testing and/or the detection of parasite-specific antibodies after the eighth month of life (when maternal antibodies disappeared).

Electrocardiography (ECG) was used as a crucial diagnostic tool to assess the disease progression during patient follow-up. No suggestive changes of chronic Chagasic cardiomyopathy were found in ECG examinations of 25 women treated before the age of 15. However, among the untreated women (n = 46), 7 of them developed typical ECG alterations such as complete right bundle branch block (n = 2) (RBBB) and left anterior fascicular block (n = 5) (LAFB) in individuals under the age of 50 [22].

### Congenital transmission

**Fig 2:**
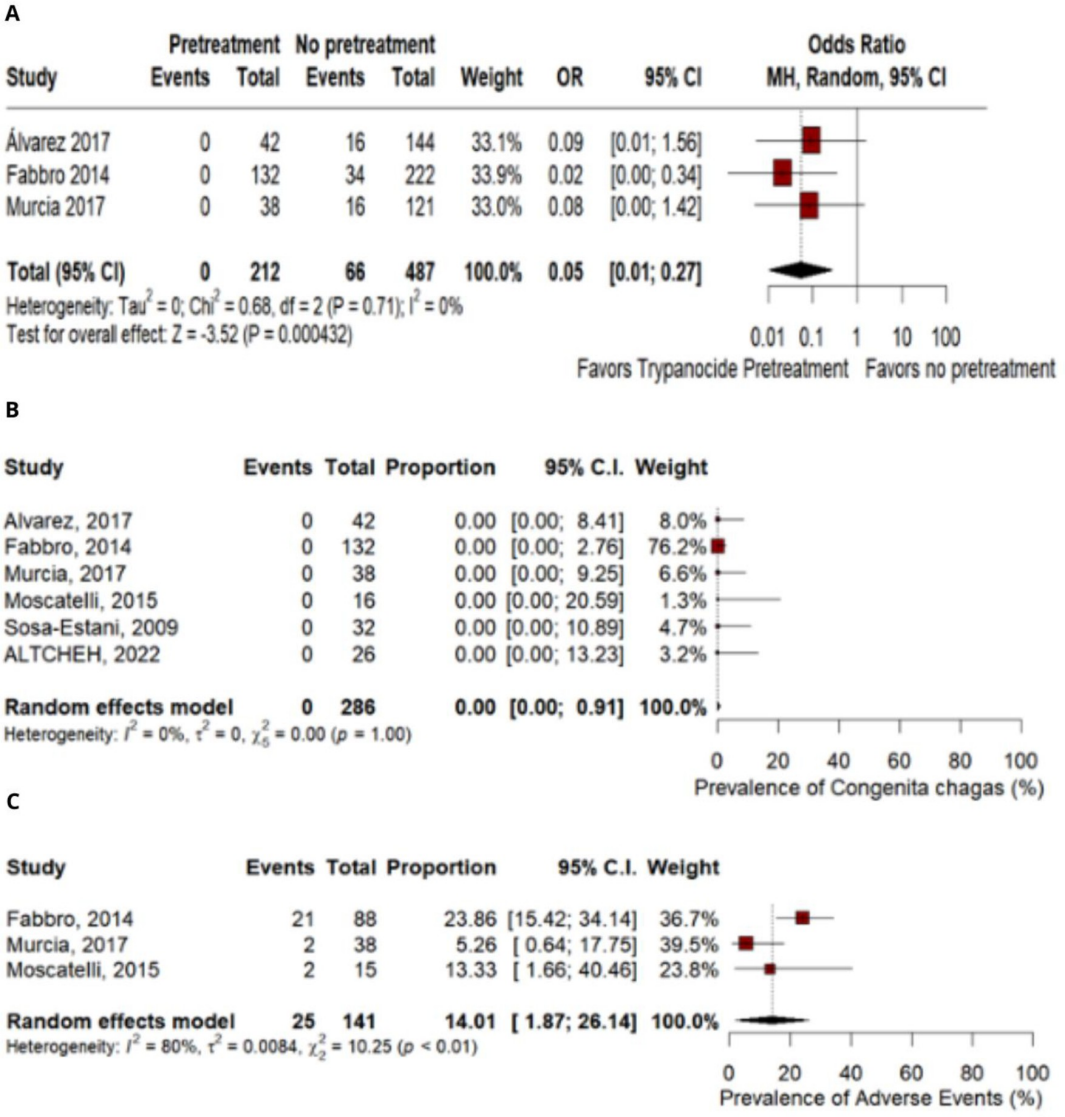
A. Prevention of mother-to-child transmission. B. Frequency of congenital Chagas in women with previous trypanocide therapy. C. Prevalence of adverse events in women used trypanocide therapy.

In children born from Tc-infected women, no transmission was observed among women who received Bzn or Nfx therapies before their pregnancy (OR 0.05; 95% Cl 0.01–0.27; p = 0.000432; I^2^ = 0%; Fig 2A) than those without a previous trypanocidal treatment.

### Prevalence of cCD transmission

All six observational studies analyzed the effectiveness of treatment outcomes in preventing congenital transmission. Among the children born from women with Chagas Disease who were previously treated with trypanocidal agents, the prevalence rate for cCD was 0.0% (95% Cl 0–0.91%; I^2^ = 0%; Fig 2B).

### Adverse events

Among all studies included, adverse events were analyzed in three studies. The rate of side effects was 14.01% (95% CI 1.87–26.14%; I^2^ = 80%; Fig 2C). There was a total of 41 AEs in a total of 141 women assessed. Overall, side effects for treatment with Benzonidazole were more prevalent than with Nifurtimox (76% vs 24%). The side effects reported in the studies were: rashes, pruritus, edema, headache, mild exanthema in the body and extremities, and gastrointestinal disorder.

### Quality assessment

The individual assessment of each observational study included in the meta-analysis is depicted in Table 2. Overall, half of the studies were deemed at higher quality, except for Murcia et al. [23], Moscatelli et al. [24], and Sosa-Estani et al. [25] were considered of lower quality, with high concerns mainly due to comparability, and exposure criteria.

**Table 2:** Critical appraisal of observational study according to the Newcastle–Ottawa Scale (NOS).

Meta-data		Methodology	Newcastle-Ottawa Scale			
Author	Publication Date	*Study design* *	*Selection*	*Comparability*	*Exposure*	*Quality*
Alvarez [21]	*2017*	*--*	☆☆☆☆	☆☆	☆☆	*8*
Fabbro [22]	*2014*	*-+*	☆☆☆☆	☆☆	☆☆☆	*9*
Murcia [23]	*2017*	*-+*	☆☆☆☆	☆	☆☆	*7*
Moscatelli [24]	*2015*	*--*	☆☆	☆	☆☆☆	*6*
Sosa-Estani [25]	*2009*	*--*	☆☆☆	☆☆	☆☆	*7*
ALTCHEH [26]	*2022*	*++*	☆☆☆☆	☆☆	☆☆	*8*

Abbreviations

*Study design: Prospective (+), Retrospective (−); single center (−), multicenter (+), Maximum quality score = 9; 0–7 points were considered lower quality, and 8–9 points were considered as higher quality

The evaluation of heterogeneity in the adverse events (AE) outcomes among women has been presented in S2 Fig. In general, heterogeneity was low in the majority of the outcomes, with a concentric distribution depicted in the funnel plots. However, the AE endpoint demonstrated an augmented heterogeneity, which was primarily attributed to the influence of Fabbro et al. [22] By funnel plot analysis, there was a slight asymmetric distribution of studies of similar weights against their standard errors. Egger’s regression test could not be performed due to the limited amount of included studies (n <10), represented on S1 and S3 Figs.

## Discussion

In this systematic review and meta-analysis of six observational studies including 1,197 patients, we confirmed the effectiveness of the preventive trypanocidal treatment of reproductive-aged women infected in reducing congenital transmission in future pregnancies. The main findings from the pooled analyses were as follows: (1) Prevention of mother-to-child transmission, (2) prevalence of cCD transmission, and (3) adverse events in women.

There has been a shift in the epidemiological profile of Chagas disease, where it has expanded from rural to urban areas and from endemic regions of Latin America to global epidemic proportions [27]. Primarily due to the advancement of globalization, with the immigration of populations from endemic areas to countries previously without the presence of the parasite *Trypanosoma cruzi*, such as Europe and the United States of America (USA) [10,27]. In endemic countries, approximately 15,000 *T*. *cruzi-infected* babies are born annually to infected mothers. The prevalence of infection in Latin American immigrants in Europe is 4.2%, and an estimated 300,000 infected immigrants are in the United States [28]. Accordingly, approximately 40,000 women of childbearing age are infected with *T*. *cruzi* in the US, which leads to an estimated 1–5% of infants born with Ccd [29–31].

Screening for congenital Chagas disease began in 2002 in addition to pre-pregnancy treatment serving as an effective preventive measure, following a recommendation by the World Health Organization (WHO) [7]. This recommendation recommended a serological test, and if positive, further testing for women at risk (women who were born in, lived in, or currently live in endemic areas) and for children born to seropositive mothers [7]. Parasitological and serological tests starting at 8 months of age with a double test were recommended (due to the presence of maternal antibodies until that age) [5,32]. In 2018, new guidelines were published by the Pan American Health Organization (PAHO), which retained some of the previous WHO recommendations but changed the timing of serological testing, recommending the use of two serological tests at the beginning of screening for women at-risk [1,31,33].

Congenital transmission average *T*. *cruzi* transmission rate from infected mothers to newborns is 4.7% [34–36]. The higher prevalence in pregnant infected women of advanced age (>30 years) reflects the trend of aging among patients with chronic Chagas disease following the successful control of vector and blood-borne transmission in recent decades [30,35,37].

Our results suggest that congenital transmission of T. cruzi could be preventable with the prior use of antitrypanocidal therapies by women of childbearing age (OR 0.05; 95% Cl 0.01–0.27; p = 0.000432). These data confirm that the use of BZD or Nfx may offer an opportunity to prevent Ccd [33,38,39]. Therefore, early screening for women in childbearing age and newborns, aiming on prompt treatment is ought to be encouraged, especially in endemic areas [8,35,40].

Among infected women who had used previous therapies, the occurrence of congenital transmission was 0.0% (95% Cl 0–0.91%). These results support previously reported data that treatment of infected women of childbearing age not only benefits the woman, but also plays an important role in preventing future congenital transmission. Therefore, in line with this, the WHO no longer focuses on diagnosing and treating infected newborns, but also on screening women of childbearing age, to detect Tc infection and treat them in order to prevent congenital transmission [8,41,42]. Therefore, working with this population is essential, according to the World Health Organization’s roadmap for neglected tropical diseases (2021–2030), for the global objective of eliminating congenital transmission worldwide by 2030 [43–49].

Adverse effects (AE) are often associated with the chosen pharmacotherapy and can have a significant impact on the overall well-being and quality of life of the patient. The majority of side effects of anti-Chagasic drugs identified were associated with benznidazole. Although the frequency of AEs was lower with nifurtimox, this medication was associated with a higher percentage of treatment-emergent AEs, leading to discontinuation. Nonetheless, the overall benefit achieved with the trypanocidal agents was significant and there were no reports of serious adverse events in the studies.

Our study has some limitations. First, the analysis was based on a restricted number of observational studies with slight differences regarding the number of patients per administered dose with 5 to 7.6 mg/kg for Benznidazole and 9.4 to 10 mg/kg for Nifurtimox. However, the low heterogeneity in the majority of outcomes suggests that our meta-analysis conveys the best available evidence for the use of trypanocidal agents in infected women of reproductive-aged to prevent cCD in infants, though there were no Randomized Controlled Trials included in this study, frailing our results. Furthermore, the absence of data did not allow for the reporting of important details, including the age range of the children and the duration of the treatement before pregancy. In addtion, other pertinent outcomes of interest, such as ECG, a detailed view of the adverse effects, and a comparison of morbidity and mortality in infected and non-infected mother-infant dyads, could not be ascertained.

Despite its limitations, our study has many strengths. Firstly, the population recruited for the studies encompassed different ethnicities from different countries and a wide range of ages. This supports the potential of the reproducibility of our results in the general population. In addition, the number of people recruited comprises a large sample per the reality of available evidence regarding cCD, and the weight of each study is similar to each other, which reduces the heterogeneity of the results or the possibility of one of the studies having more influence on the final result.

## Conclusion

Our meta-analysis confirms that the treatment of Tc-infected women of reproductive age is significantly associated with the prevention of congenital Tc infection. Our results support the idea that the administration of benzonidazole or nifurtimox to women of reproductive age should be encouraged to prevent congenital transmission.

## Supporting information

S1 Table PRISMA 2020 Checklist. (DOCX)

S2 Table PRISMA 2020 for Abstract Checklist. (DOCX)

S3 Table Search strategies. (DOCX)

S4 Table Trypanocidal drugs included in the meta-analysis. (DOCX)

S1 Fig Funnel Prevention of mother-to-child transmission. (TIF)

S2 Fig Heterogeneity for Adverse Events in mothers. (TIF)

S3 Fig Funnel frequency of vertical transmission. (TIF)
