# Supplementary material for: Prevention of congenital chagas disease by trypanocide treatment in women of reproductive age: A meta-analysis of observational studies
Source: PLoS Negl Trop Dis. 2024 Sep 5;18(9):e0012407. doi: 10.1371/journal.pntd.0012407 (PMC11376591; doi:10.1371/journal.pntd.0012407)
Supplement: S3 Table — (DOCX) [file pntd.0012407.s003.docx]

**Supplementary Table 3 Search strategies.**

| **Database** | **Search Strategy** |
| --- | --- |
| **PubMed** | (“Chagas Disease”[MeSH Terms] OR “Chagas Disease”[Title/Abstract] OR “Pregnancy complications, parasitic”[MeSH Terms] OR “female”[Title/Abstract] OR “women”[Title/Abstract] OR “pregnancy”[Title/Abstract] OR “pregnancy”[MeSH Terms] OR “childbearing age”[Title/Abstract] OR "reproductive age"[Title/Abstract] OR "infected woman"[Title/Abstract]) AND ("benznidazol"[Title/Abstract] OR "nifurtimox"[Title/Abstract] OR "treatment"[Title/Abstract] OR "therapy"[Title/Abstract] OR "trypanocide"[Title/Abstract] AND “congenital chagas”[Title/Abstract] OR "vertical transmission"[Title/Abstract]) |
| **Scopus** | (TITLE-ABS-KEY ( "benznidazole" ) OR TITLE-ABS-KEY ( "nifurtimox" ) OR TITLE-ABS-KEY ( "treatment" ) OR TITLE-ABS-KEY ( "therapy" ) OR TITLE-ABS-KEY ( "trypanocide" ) AND TITLE-ABS-KEY ( "chagas disease" ) OR TITLE-ABS-KEY ( "women" ) OR TITLE-ABS-KEY ( "childbearing age" ) OR TITLE-ABS-KEY ( "reproductive age" ) AND TITLE-ABS-KEY ( "congenital chagas" ) OR TITLE-ABS-KEY ( "vertical transmission" ) ) |
| **Web of Science** | (TS=("chagas disease") OR TS=(trypanosomiasis) OR TS=(adult) OR TS=(woman) OR TS=(female) OR TS=("childbearing age") OR TS=("reproductive age") OR TS=(pregnant) OR TS=(pregnancy)) AND (TS=(benznidazol) OR TS=(benzonidazol) OR TS=(nifurtimox) OR TS=("trypanocide therapy") AND TS=(congenital) OR TS=("vertical transmission" OR TS=(control) OR TS=(controle)) |
| **LILACS** | ((chagas) OR (mujer) OR (mulher) OR (gravida) OR (embarazadas) OR (idade reprodutiva) OR (edad reproductiva)) AND ((benznidazol) OR (benzonidazol) OR (nifurtimox)) AND ((congênito) OR (congênita) OR (prevenção) OR (prevencion) OR (control) OR (controle)) |
| **The Cochrane Library** | (("chagas disease"):ti,ab,kw OR (trypanosomiasis):ti,ab,kw OR (adult):ti,ab,kw OR (woman):ti,ab,kw OR (female):ti,ab,kw OR ("childbearing age"):ti,ab,kw OR ("reproductive age"):ti,ab,kw OR (pregnant):ti,ab,kw OR (pregnancy):ti,ab,kw) AND ((benzonidazole):ti,ab,kw OR (benznidazol):ti,ab,kw OR (nifurtimox):ti,ab,kw OR ("trypanocide therapy"):ti,ab,kw AND (congenital):ti,ab,kw OR ("vertical transmission"):ti,ab,kw kw OR (prevention):ti,ab,kw OR (control):ti,ab,kw) |
