# Supplementary material for: Prevention of congenital chagas disease by trypanocide treatment in women of reproductive age: A meta-analysis of observational studies
Source: PLoS Negl Trop Dis. 2024 Sep 5;18(9):e0012407. doi: 10.1371/journal.pntd.0012407 (PMC11376591; doi:10.1371/journal.pntd.0012407)
Supplement: S4 Table — (DOCX) [file pntd.0012407.s004.docx]

**Supplementary Table 4** Trypanocidal drugs included in the meta-analysis.

| **Compound** | **Chemical structure** | **Molecular Formula** | **AUC** | **Recommended dose** | **Absorption** | **Elimination** | **Terminal half-life** |
| --- | --- | --- | --- | --- | --- | --- | --- |
| Benznidazole | 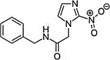 | C_12_H_12_N_4_O_3_ | 46.4 μgml/h | 5 to 7.5 mg/kg per day for 60-90 days | Gastrointestinal tract | Renal | 12.1h |
| Nifurtimox | 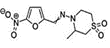 | C_10_H_13_N_3_O_5_S | 5.43 ngml/h | 8 to 10 mg/kg per day for 60-90 days | Gastrointestinal tract | Hepatic and renal | 2.95h |
