# Supplementary figures and images for: Prevention of congenital chagas disease by trypanocide treatment in women of reproductive age: A meta-analysis of observational studies
Source: PLoS Negl Trop Dis. 2024 Sep 5;18(9):e0012407. doi: 10.1371/journal.pntd.0012407 (PMC11376591; doi:10.1371/journal.pntd.0012407)

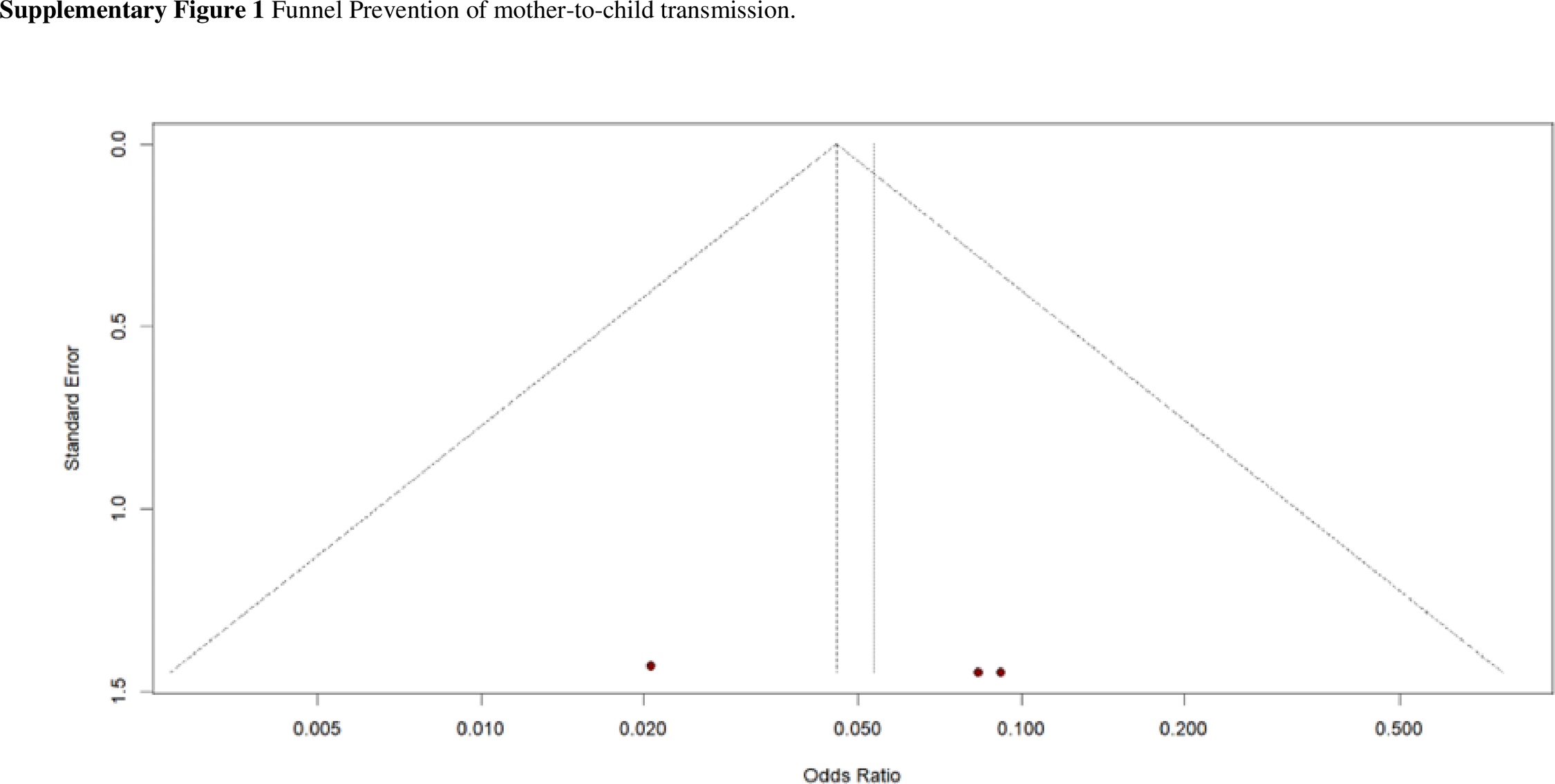

Supplement: S1 Fig — (TIF) [file pntd.0012407.s005.tif]

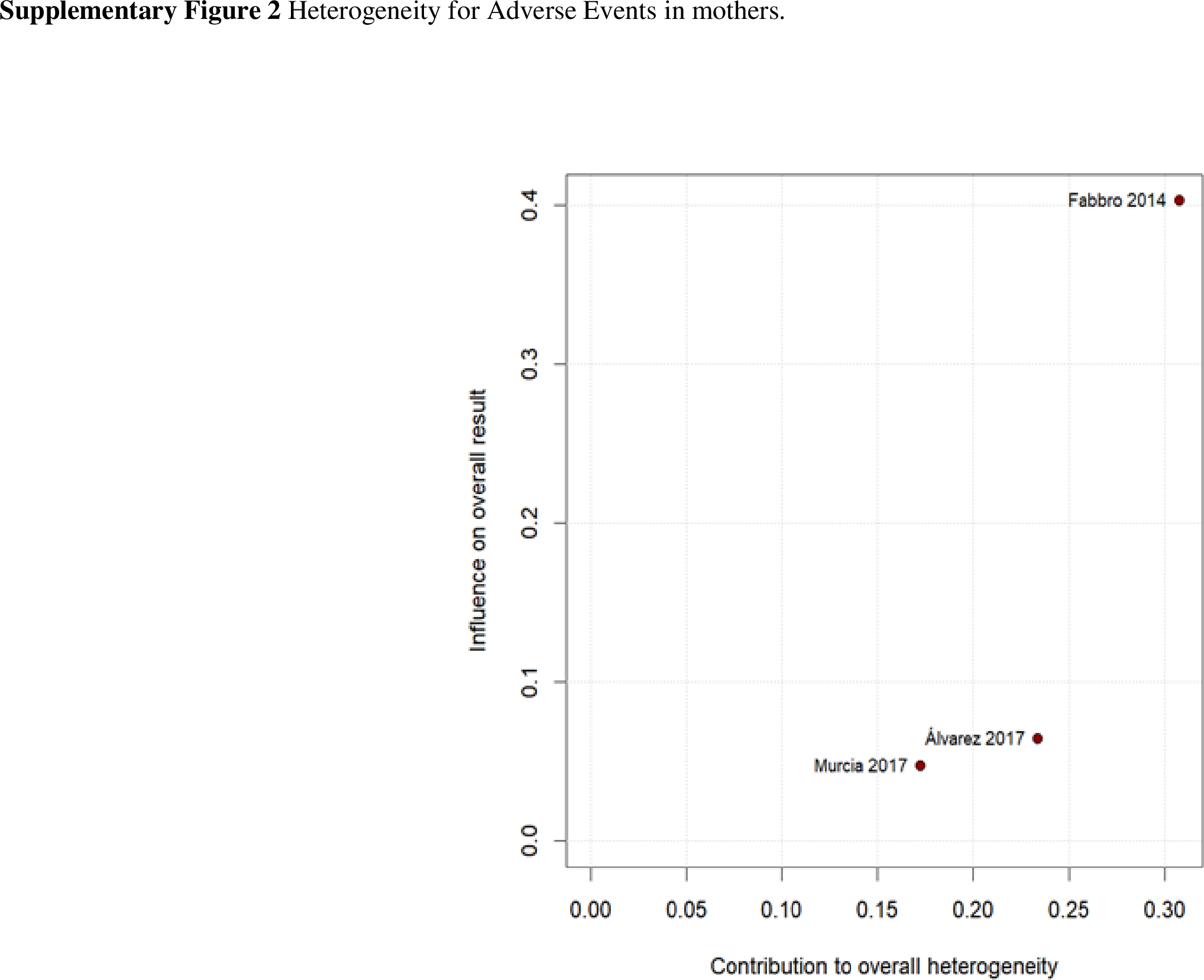

Supplement: S2 Fig — (TIF) [file pntd.0012407.s006.tif]

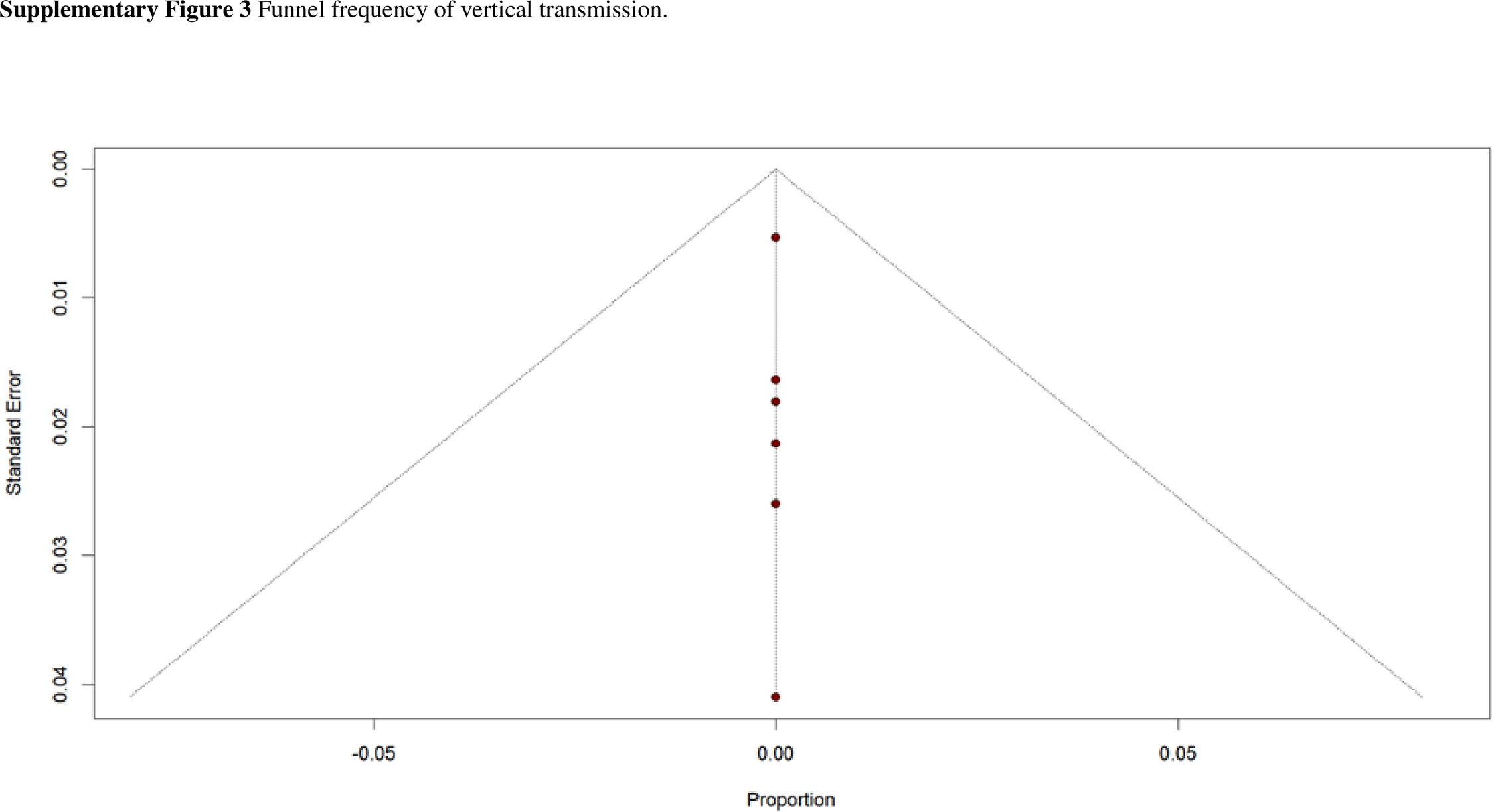

Supplement: S3 Fig — (TIF) [file pntd.0012407.s007.tif]
